# Early childhood neurodevelopmental milestones in children with allergic diseases: the Japan Environment and Children’s Study (JECS)

**DOI:** 10.1038/s41598-024-57210-y

**Published:** 2024-03-18

**Authors:** Abir Nagata, Kazunari Onishi, Toshio Masumoto, Takatoshi Nakagawa, Kazue Ishitsuka, Youichi Kurozawa

**Affiliations:** 1 Department of Regenerative Dermatology, Graduate School of Medicine, Osaka University, 2-2, Yamadaoka, Suita-shi, Osaka, 565-0871 Japan; 2 Graduate School of Public Health, St. Luke’s International University, Tokyo, Japan; 3 Division of Environmental Health, Graduate School of Public Health, St. Luke’s International University, 3-6-2 Tsukiji, Chuo-ku, Tokyo, 104-0045 Japan; 4 Division of Health Administration and Promotion, Faculty of Medicine, Tottori University, Tottori, Japan; 5 Department of Social Medicine, National Center for Child Health and Development, Tokyo, Japan

**Keywords:** Epidemiology, Paediatric research, Neurodevelopmental disorders, Paediatric neurological disorders

## Abstract

This study investigated the potential link between early childhood allergic diseases and neurodevelopmental milestone attainment during the first 3 years of life. Utilizing data from a large-scale prospective birth cohort study in Japan, encompassing 87,986 children, we examined physician-diagnosed and caregiver-reported allergic conditions, including atopic dermatitis (AD), asthma, and food allergy (FA). Neurodevelopmental milestones were assessed using the Ages and Stages Questionnaires at 1, 1.5, 2, 2.5, and 3 years of age. Stabilized inverse probability-weighted generalized estimating equation models were employed to estimate odds ratios (ORs). Our analysis revealed no significant association of AD and asthma with delay in communication, gross motor, fine motor, problem-solving, and personal-social skills during the initial 3 years of life. However, children with FA showed an increased likelihood of experiencing gross motor delay compared with that shown by those without FA (weighted adjusted OR: 1.14). Despite this, no significant association of FA with other developmental domains was observed. Early childhood allergies may not influence neurodevelopmental delays. However, there is a potential association between FA and delays, specifically in gross motor skills, that warrants routine developmental monitoring and additional investigations.

## Introduction

Allergic diseases—such as atopic dermatitis (AD), asthma, and food allergy (FA)—are prevalent chronic disorders marked by immune system dysregulation and commonly manifest during the early stages of infancy^1,2^. These conditions impose a significant health burden, impact the well-being of both caregivers and children, affect sleep quality, and contribute to psychiatric, behavioral, and neurodevelopmental disorders (NDDs)^3–6^. Allergic diseases and NDDs are multifactorial etiopathogenetic processes that result from complex interactions between genetic and environmental factors and are accompanied by shared phenomena such as immune dysregulation and inflammation^7,8^. However, it remains unclear whether specific allergic conditions can adversely influence early neurodevelopmental milestones.

Recently, the increasing prevalence of early childhood allergic diseases has coincided with an increase in childhood-onset NDDs^8,9^. Consequently, numerous studies have focused on exploring the connection between the immune and nervous systems, particularly during early infancy. A proposed mechanism is that allergic diseases are triggered by an acute immune response, resulting in chronic inflammation and the activation of basophils, mast cells, and eosinophils through allergen-induced cross-linking with immunoglobulin E^7,10–12^. Subsequently, these conditions activate an immediate hypersensitivity response, leading to elevated levels of inflammatory mediators, including cytokines or eosinophils, which have the potential to disrupt central nervous system homeostasis, thereby leading to neurological, cognitive, and motor function disorders in the affected offspring^7,10–12^. Additionally, varying degrees of NDDs and impaired cognitive and motor function in children with allergic conditions, particularly AD, asthma, and FA, have been reported^9,11,13–16^. Conversely, a lesser likelihood of cognitive dysfunction being associated with allergic conditions has been suggested^17,18^. Further, not all allergies have been equally linked to neurodevelopmental dysfunctions^11,18–20^.

Despite the high prevalence of allergic diseases in Japan^21,22^, data on early childhood allergic conditions and their potential impact on neurodevelopment are scarce. Herein, we aimed to investigate the associations of AD, asthma, and FA with neurodevelopmental milestones (communication, gross motor, fine motor, problem-solving, and personal-social skills) in Japanese children using the data derived from the Japan Environment and Children’s Study (JECS), an ongoing prospective birth cohort study in Japan.

## Results

### Cohort characteristics

**Figure 1:**
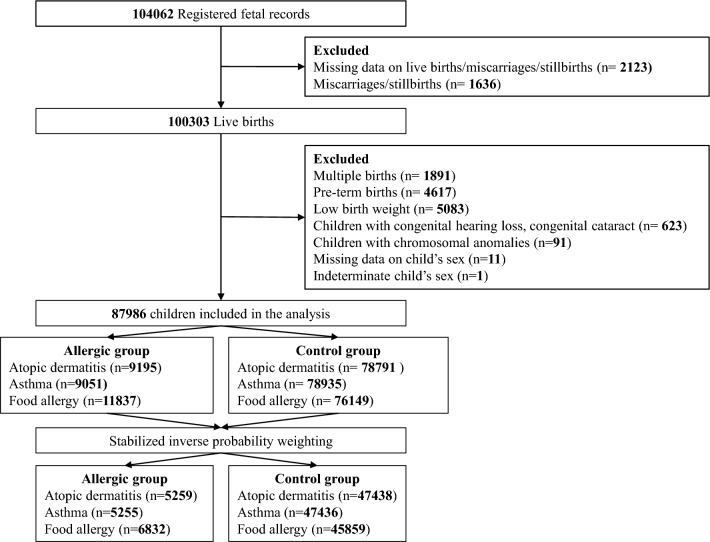
Flowchart of the participant selection process.

Characteristics of the study participants are depicted in Table 1; 87,986 individuals were included in the study (boys, n = 45,357 [51.6%]; girls, n = 42,629 [48.4%]). Of these, 9195, 9051, and 11,837 reported having AD, asthma, and FA, respectively (Fig. 1). Among the mothers, 13,738 (15.6%), 9486 (10.8%), and 4187 (4.8%) had a history of pre-pregnancy AD, asthma, and FA, respectively (Table 1). Supplementary Tables S1 and S2 summarize the children’s developmental status according to the allergic conditions. Among children aged 1 and 2 years, 3883 (4.4%) and 3819 (4.3%) had suspected delays in gross motor skills, respectively.

**Table 1:** Distribution of participant characteristics according to allergic diseases.

Participants characteristics		Allergic diseases, No. (%)		
	Total (n = 87,986)	Atopic dermatitis (n = 9195)	Asthma (n = 9051)	Food allergy (n = 11,837)
Maternal and pregnancy characteristics				
Age at delivery (years)	No. (%)			
< 25	7024 (8.0)	667 (9.1)	794 (11.0)	780 (8.2)
25–29	19,010 (21.6)	2106 (28.6)	2123 (29.6)	2889 (30.3)
30–34	24,399 (27.7)	2676 (36.3)	2487 (34.7)	3439 (36.1)
≥ 35	18,584 (21.1)	1921 (26.0)	1770 (24.7)	2425 (25.4)
Missing data	18,969 (21.6)	NA	NA	NA
Marital status				
Single	3021 (3.4)	283 (3.1)	289 (3.2)	347 (3.0)
Married	82,792 (94.1)	8748 (96.0)	8552 (95.5)	11,311 (96.4)
Divorced/widowed	707 (0.8)	78 (0.9)	114 (1.3)	79 (0.6)
Missing data	1466 (1.7)	NA	NA	NA
Education				
High school or less	31,235 (35.5)	3123 (34.3)	3353 (37.6)	3712 (31.6)
College	36,124 (41.1)	3799 (41.8)	3878 (43.4)	5077 (43.4)
Bachelor’s degree or more	18,768 (21.3)	2178 (23.9)	1697 (19.0)	2922 (25.0)
Missing data	1859 (2.1)	NA	NA	NA
Pre-pregnancy BMI (kg/m^2^)				
< 18.5	13,784 (15.7)	1468 (16.4)	1368 (15.5)	1876 (16.2)
18.5–24.9	62,880 (71.5)	6676 (74.4)	6489 (73.6)	8728 (75.6)
≥ 25	8390 (9.5)	831 (9.2)	963 (10.9)	948 (8.2)
Missing data	2932 (3.3)	NA	NA	NA
Gestational diabetes				
No	85,414 (97.1)	8928 (97.3)	8787 (97.3)	11,522 (97.5)
Yes	2291 (2.6)	244 (2.7)	240 (2.7)	290 (2.5)
Missing data	281 (0.3)	NA	NA	NA
Infertility treatment				
No	82,104 (93.3)	8594 (93.9)	8494 (94.2)	10,935 (92.9)
Yes	5438 (6.2)	555 (6.1)	520 (5.8)	841 (7.1)
Missing data	444 (0.5)	NA	NA	NA
Psychological distress (K6 scale)^a^				
0–4	58,940 (67.0)	5951 (65.3)	5832 (65.0)	7791 (66.4)
> 5 (distress)	27,606 (31.4)	3160 (34.7)	3134 (35.0)	3944 (33.6)
Missing data	1440 (1.6)	NA	NA	NA
Iron pill				
No	49,839 (56.6)	5244 (57.2)	5044 (55.9)	6684 (56.6)
Yes	37,866 (43.0)	3928 (42.8)	3983 (44.1)	5128 (43.4)
Missing data	281 (0.4)	NA	NA	NA
Folic acid				
No	85,922 (97.7)	8994 (98.1)	8866 (98.2)	11,568 (97.9)
Yes	1783 (2.0)	178 (1.9)	161 (1.8)	244 (2.1)
Missing data	281 (0.3)	NA	NA	NA
Smoking during pregnancy				
No	82,177 (93.4)	8720 (96.0)	8396 (94.2)	11,282 (96.5)
Yes	3718 (4.2)	362 (4.0)	516 (5.8)	412 (3.5)
Missing data	2091 (2.4)	NA	NA	NA
Alcohol during pregnancy				
No	83,450 (94.8)	8822 (97.3)	8619 (97.0)	11,392 (97.5)
Yes	2431 (2.8)	248 (2.7)	271 (3.0)	296 (2.5)
Missing data	2105 (2.4)	NA	NA	NA
Atopic dermatitis				
No	73,150 (83.1)	6557 (71.7)	7368 (81.9)	8869 (75.3)
Yes	13,738 (15.6)	2582 (28.3)	1625 (18.1)	2902 (24.7)
Missing data	1098 (1.3)	NA	NA	NA
Asthma				
No	77,402 (88.0)	7792 (85.3)	7203 (80.1)	10,093 (85.7)
Yes	9486 (10.8)	1347 (14.7)	1790 (19.9)	1678 (14.3)
Missing data	1098 (1.2)	NA	NA	NA
Food allergy				
No	82,701 (94.0)	8441 (92.4)	8398 (93.4)	10,866 (92.3)
Yes	4187 (4.8)	698 (7.6)	595 (6.6)	905 (7.7)
Missing data	1098 (1.2)	NA	NA	NA
Household characteristics				
Paternal education				
High school or less	37,682 (42.8)	3902 (43.2)	4215 (47.5)	4728 (40.6)
College	19,254 (21.9)	2082 (23.0)	2072 (23.4)	2709 (23.2)
Bachelor’s degree or more	28,635 (32.5)	3061 (33.8)	2581 (29.1)	4224 (36.2)
Missing data	2415 (2.8)	NA	NA	NA
Paternal history of smoking				
No	45,380 (51.6)	4900 (54.5)	4431 (50.3)	6524 (56.3)
Yes	39,630 (45.0)	4094 (45.5)	4374 (49.7)	5066 (43.7)
Missing data	2976 (3.4)	NA	NA	NA
Income (million Japanese yen/year)				
< 4	32,336 (36.8)	3452 (40.1)	3627 (43.5)	4240 (38.2)
4–8	39,449 (44.8)	4219 (49.0)	3885 (46.5)	5630 (50.7)
≥ 8	8690 (9.9)	936 (10.9)	835 (10.0)	1233 (11.1)
Missing data	7511 (8.5)	NA	NA	NA
Child characteristics				
Mode of delivery				
Cesarean	14,664 (16.7)	1515 (16.6)	1572 (17.5)	1879 (16.0)
Vaginal	72,818 (82.7)	7635 (83.4)	7427 (82.5)	9898 (84.0)
Missing data	504 (0.6)	NA	NA	NA
Child’s sex				
Boy	45,357 (51.6)	5308 (57.7)	5484 (60.6)	6931 (58.6)
Girl	42,629 (48.4)	3887 (42.3)	3567 (39.4)	4906 (41.4)
Gestational age (weeks)				
37–38	27,434 (31.2)	2886 (31.5)	3087 (34.2)	3478 (29.4)
39–41	60,051 (68.3)	6268 (68.3)	5918 (65.6)	8302 (70.3)
> 41	220 (0.2)	18 (0.2)	22 (0.2)	32 (0.3)
Missing data	281 (0.3)	NA	NA	NA
Birth weight (g)				
2500–4000	86,816 (98.7)	9057 (98.8)	8924 (98.9)	11,676 (98.9)
> 4000	831 (0.9)	108 (1.2)	95 (1.1)	128 (1.1)
Missing data	339 (0.4)	NA	NA	NA
Breastfeeding				
0–6 months				
No	5154 (5.9)	523 (5.8)	669 (7.6)	506 (4.4)
Yes	77,029 (87.5)	8477 (94.2)	8179 (92.4)	11,107 (95.6)
Missing data	5803 (6.6)	NA	NA	NA
7–12 months				
No	20,083 (22.8)	2053 (23.0)	2673 (30.5)	2141 (18.5)
Yes	59,459 (67.6)	6876 (77.0)	6090 (69.5)	9416 (81.5)
Missing data	8444 (9.6)	NA	NA	NA
Formula feeding				
0–6 months				
No	46,175 (52.5)	5368 (59.6)	4572 (51.7)	7516 (64.7)
Yes	36,008 (40.9)	3632 (40.4)	4276 (48.3)	4097 (35.3)
Missing data	5803 (6.6)	NA	NA	NA
7–12 months				
No	39,236 (44.6)	4618 (51.7)	3695 (42.2)	6500 (56.2)
Yes	40,306 (45.8)	4311 (48.3)	5068 (57.8)	5057 (43.8)
Missing data	8444 (9.6)	NA	NA	NA
Nursery attendance at 1 year old				
No	57,764 (65.7)	6189 (69.6)	5155 (59.3)	8141 (70.7)
Yes	21,434 (24.3)	2697 (30.4)	3544 (40.7)	3367 (29.3)
Missing data	8788 (10.0)	NA	NA	NA

### Association of early childhood allergic diseases with neurodevelopmental outcomes

Table 2 depicts the outcomes of the stabilized inverse probability-weighted (sIPW) generalized estimating equation (GEE) models, which assessed the overall association between each allergic condition and Ages and Stages Questionnaires, third edition (ASQ-3) developmental milestones during the initial 3 years of age. Our analysis revealed no significant associations of AD with communication, gross motor, fine motor, problem-solving, and personal-social skills delay. These findings remained consistent even after adjusting for other allergic conditions. Compared with children without asthma, children with asthma had lower odds of experiencing delayed development in communication skills (weighted adjusted odds ratio [aOR]: 0.77; 95% confidence interval [CI] 0.68–0.88; *p* = 0.002). Notably, children with FA exhibited a heightened likelihood of experiencing gross motor delay than that observed with those without FA (weighted aOR: 1.14; 95% CI 1.04–1.24; *p* = 0.003). The E-value for the association between FA and gross motor skills delay was 1.54, with a 95% CI value of 1.28; thus, any unmeasured confounders must have a risk ratio association with both FA and gross motor skills exceeding 1.28 to fully explain the observed association. No substantial associations of asthma and FA with the remaining developmental milestones were observed.

**Table 2:** Association of allergic diseases with ASQ-3 developmental milestones.

Allergic diseases	Child, No. (%)^a^	ASQ-3 domains OR (95% CI)									
		Communication skills		Gross motor skills		Fine motor skills		Problem-solving skills		Personal-social skills	
Atopic dermatitis		Model 1^b^	Model 2^c^	Model 1^b^	Model 2^c^	Model 1^b^	Model 2^c^	Model 1^b^	Model 2^c^	Model 1^b^	Model 2^c^
No	47,438 (90.0)	1 [Reference]	1 [Reference]	1 [Reference]	1 [Reference]	1 [Reference]	1 [Reference]	1 [Reference]	1 [Reference]	1 [Reference]	1 [Reference]
Yes	5259 (10.0)	1.01 (0.90–1.15)	1.03 (0.90–1.19)	1.00 (0.92–1.09)	0.94 (0.84–1.04)	1.04 (0.96–1.13)	1.04 (0.95–1.14)	1.04 (0.96–1.12)	1.03 (0.94–1.13)	0.97 (0.85–1.10)	0.93 (0.90–1.08)
Asthma											
No	47,436 (90.0)	1 [Reference]	1 [Reference]	1 [Reference]	1 [Reference]	1 [Reference]	1 [Reference]	1 [Reference]	1 [Reference]	1 [Reference]	1 [Reference]
Yes	5255 (10.0)	0.78 (0.68–0.89)^d^	0.77 (0.68–0.88)^d^	0.99 (0.91–1.08)	0.98 (0.90–1.07)	0.98 (0.90–1.08)	0.97 (0.89–1.05)	0.95 (0.83–1.02)	0.93 (0.81–1.04)	0.88 (0.77–1.03)	0.86 (0.73–1.02)
Food allergy											
No	45,859 (87.0)	1 [Reference]	1 [Reference]	1 [Reference]	1 [Reference]	1 [Reference]	1 [Reference]	1 [Reference]	1 [Reference]	1 [Reference]	1 [Reference]
Yes	6238 (13.0)	0.96 (0.87–1.07)	0.95 (0.84–1.08)	1.12 (1.04–1.21)^d^	1.14 (1.04–1.24)^d^	1.05 (0.98–1.12)	1.02 (0.93–1.11)	1.02 (0.96–1.10)	1.01 (0.93–1.09)	1.04 (0.93–1.15)	1.07 (0.94–1.02)

OR, Odds ratio; CI, confidence interval; ASQ-3, Ages and Stages Questionnaires, third edition.

^a^Data are expressed as weighted No. (%).

^b^Weighted OR. Weighted OR were derived via propensity score-based, stabilized inverse probability-weighted generalized estimating equations models.

^c^Weighted adjusted OR; adjusted for other allergic diseases.

^d^*p* < 0.01.

Table 3 presents the results of the GEE models, examining the association of allergic persistence with ASQ-3 developmental milestones. Children with intermittent or persistent AD demonstrated no significant associations with any of the five developmental domains during 3 years of age. In contrast to children without asthma, children with intermittent asthma had lower odds of experiencing delayed development in communication skills (aOR: 0.85; 95% CI 0.75–0.96; *p* = 0.007). Children with intermittent FA were associated with gross motor delays (aOR: 1.16; 95% CI 1.06–1.27; *p* = 0.009). Conversely, no significant associations were found between persistent asthma, FA, and all developmental domains. Furthermore, no associations were detected between International Study of Asthma and Allergies in Childhood (ISAAC)-based, eczema/AD, and wheezing features and delayed ASQ-3 milestones (Supplementary Table S3) at 3 years of age.

**Table 3:** Association of allergic disease persistence with ASQ-3 developmental milestones.

Disease persistence^a^	Child, No. (%)	ASQ-3 domains, AOR (95% CI)				
		Communication skills	Gross motor skills	Fine motor skills	Problem-solving skills	Personal-social skills
Atopic dermatitis^b^						
None	78,791 (89.5)	1 [Reference]	1 [Reference]	1 [Reference]	1 [Reference]	1 [Reference]
Intermittent	7921 (9.0)	1.00 (0.88–1.15)	0.94 (0.86–1.02)	1.02 (0.94–1.11)	1.04 (0.96–1.12)	0.94 (0.83–1.07)
Persistent	1274 (1.5)	1.24 (0.93–1.67)	1.04 (0.86–1.24)	1.07 (0.88–1.29)	1.04 (0.87–1.24)	1.14 (0.87–1.50)
Asthma^b^						
None	78,935 (89.7)	1 [Reference]	1 [Reference]	1 [Reference]	1 [Reference]	1 [Reference]
Intermittent	8456 (9.6)	0.85 (0.75–0.96)^c^	0.99 (0.92–1.07)	0.96 (0.89–1.04)	0.93 (0.87–1.00)	0.90 (0.81–1.01)
Persistent	595 (0.7)	0.94 (0.61–1.41)	1.21 (0.93–1.58)	1.02 (0.81–1.29)	0.83 (0.65–1.05)	1.08 (0.75–1.53)
Food allergy^b^						
None	76,149 (86.5)	1 [Reference]	1 [Reference]	1 [Reference]	1 [Reference]	1 [Reference]
Intermittent	11,149 (12.7)	0.99 (0.88–1.12)	1.16 (1.06–1.27)^d^	1.04 (0.96–1.12)	1.05 (0.98–1.13)	1.06 (0.95–1.18)
Persistent	688 (0.8)	1.19 (0.81–1.74)	1.07 (0.83–1.38)	1.16 (0.92–1.47)	1.12 (0.90–1.40)	1.06 (0.73–1.53)

AOR was derived via generalized estimating equations models (adjusted for maternal age at delivery, marital status, pre-pregnancy body mass index, gestational diabetes, infertility treatment, psychological distress, iron and folic acid supplementation, pre-pregnancy history of AD, asthma, FA, alcohol consumption, maternal and paternal formal education, smoking during pregnancy, household income, mode of delivery, child's sex, gestational age, birth weight, breastfeeding, formula feeding, and child’s nursery attendance).

AD, Atopic dermatitis; FA, food allergy; AOR, adjusted odds ratio; CI, confidence interval; ASQ-3, Ages and Stages Questionnaires, third edition.

^a^Persistence of allergies were based on the caregiver’s responses at child’s age 1, 1.5, 2 and 3 years. (None, no specific allergy at any survey points; Intermittent, specific allergy at either 1, 2 or 3 survey points; and Persistent, specific allergy at all survey points).

^b^Additionally adjusted for other allergic diseases.

^c^*p* < 0.01.

^d^*p* < 0.001.

### Sensitivity and subgroup analyses

The results from the following sensitivity analyses, specifically models with (a) trimmed extreme weights, (b) multiple imputations, (c) excluded missing values, and (d) present sample means—(2 × standard deviation [SD]) as cutoff values for each ASQ-3 domain, were in concordance with those from the main analyses (Supplementary Tables S4–S7). Regarding the analysis of allergic comorbidities, no associations were identified between any allergy, AD with asthma, and AD with FA and delay in any of the five developmental milestones. However, asthma with FA and all the three allergies were associated with gross motor skills delay (aOR: 1.37; 95% CI 1.13–1.66; *p* < 0.001; aOR: 1.20; 95% CI 1.01–1.43; *p* = 0.045, respectively; Supplementary Table S8). Furthermore, no evidence suggested an association between each allergic disease and the ASQ-3 developmental milestones with child’s sex as a potential modifying factor (all *p* values for interaction > 0.05; Supplementary Table S9).

## Discussion

In this cohort, we evaluated the association between allergic diseases (AD, asthma, FA) and communication, gross motor, fine motor, problem-solving, and social functioning skills in the initial 3 years of life. Our findings showed no significant association between AD, asthma (including cases with intermittent or persistent conditions), and these developmental skills, except for a higher risk of gross motor delay in children with FA than that in those without FA. These findings were consistent across sensitivity analyses. Collectively, our findings indicate the absence of substantial neurodevelopmental delays caused by allergic diseases during early childhood.

Our findings contradict those of existing studies that reported a positive link between childhood allergy and neurodevelopment. Consistent with our findings, a cross-sectional study found no link between allergic diseases and children’s cognitive functions^17^. Bakkaloglu et al. reported that allergic features were not frequent in children with autism spectrum disorders (ASD)^23^. A previous meta-analysis^19^ and a longitudinal birth cohort study found no association between asthma and increased likelihood of ASD^20^. In this study, we observed an association of asthma with a lower likelihood of communication skills delay. However, this association should not be interpreted as a protective effect of asthma on the development of communication skills. One possible explanation for this finding is that children with asthma may benefit from more frequent healthcare visits, facilitating the early identification and management of developmental delays^24^. Additionally, the proactive approach of parents in managing chronic conditions like asthma can lead to more effective support and management of their child’s health^24,25^.

Several observational studies have reported an association of childhood allergy with ASD and attention-deficit/hyperactivity disorder (ADHD)^9,26–28^. Zaitsu et al. reported that Japanese children with developmental disorders are more likely to have allergic diseases^29^. Previous reports have suggested the presence of a strong link between AD and cognitive, behavioral, and neurodevelopmental dysfunctions^6,18,30,31^. However, our findings demonstrated the absence of associations between AD alone and neurodevelopmental delays. Consistent with our findings, a birth cohort study found no associations between preschool eczema and ADHD^32^. Furthermore, Rodriguez et al., reported no strong connection between IgE-mediated atopy and neurodevelopmental outcomes^33^.

The observed heterogeneities among these studies may be attributed to variations in the study settings, duration of follow-up, sample sizes, types of allergies investigated, and assessment tools used for neurodevelopmental evaluation. However, previous studies mainly focused on determining the prevalence of specific conditions such as ASD or ADHD in children who had already received an appropriate diagnosis or were registered in relevant databases^9,27,28,34^. Herein, we applied the ASQ-3, and although it may not fully capture the intricacies of ASD, it has a diagnostic accuracy exceeding 80% and effectively identifies ASD in most cases^35,36^. The co-occurrence of allergies and developmental conditions does not necessarily imply a direct causal relationship. It is plausible that the development of communication, motor, problem-solving, and social skills involves complex interactions within the brain^37^, and the inflammation associated with allergic diseases may not directly influence the specific regions responsible for these abilities. Further, allergic diseases may indirectly impact developmental skills through factors related to allergic symptoms and their management. For instance, discomfort resulting from allergies and disrupted sleep patterns may temporarily affect focus and engagement as well as have short-term effects on cognitive function^38,39^.

Evidence suggests that FA disrupts brain function, leading to emotional and behavioral disorders as well as NDDs^27,28,40,41^. However, inconsistencies have also been observed across studies^11,16^. We found that most developmental domains showed no associations with FA, except for those responsible for gross motor skills. A plausible explanation is that FA symptoms, such as itching, swelling, hives, digestive issues, or respiratory problems, may indirectly impair a child’s gross motor skills by causing discomfort, pain, or fatigue, thereby affecting their physical performance and activity engagement^42–45^. Furthermore, if a child experiences digestive issues or discomfort due to FA, it may lead to dietary and social limitations and decreased participation in physical activity, which can indirectly affect gross motor skills^42–45^. The observed higher risk of gross motor skills delay was exclusively found in children with FA, notably among those with intermittent FA. However, it is important to highlight that only 688 (8%) of the 11,837 FA cases were reported to have persistent FA, which may have led to an underestimation of the observed association. Nevertheless, it is essential to implement measures that aim at preventing and detecting gross motor delays in children with food allergies.

We observed no sex differences in the association between allergic diseases and neurodevelopment. Additionally, no association was observed between AD plus asthma and AD plus FA with neurodevelopment. However, we found an association when asthma and FA co-occurred (including when AD was also present) with gross motor skills, suggesting that this combination might exert a more systemic impact on a child's health^46^. This impact could influence neurodevelopment or increase the psychological stress from managing multiple chronic conditions. Further studies are necessary to elucidate the interactions and potential connections between genetic and environmental factors in influencing neurodevelopmental outcomes.

### Strengths and limitations

Our study, utilizing a large and reliable Japanese birth cohort dataset that represents the Japanese population^47^, provides unprecedented insights into the connection between childhood allergy and neurodevelopmental milestones. It marks an advancement in diversifying the research on immune disturbances and neurodevelopmental variability across different geographical and ethnic groups. By focusing on the Japanese population, this research does not only enrich the existing literature, primarily based on Western cohorts, but also highlights the potential for distinctive genetic and environmental interplays in Eastern settings. Furthermore, we included repeatedly-measured developmental milestone data and a wide range of confounding factors that few previous studies have accounted for. However, the study has some limitations. First, data on both allergic diseases and ASQ-3 were based on parental reporting, potentially causing reporting bias and non-differential misclassifications, obscuring true associations. Second, we lacked data on several key aspects including the timing of onset and severity of allergic conditions, medication usages, laboratory parameters, and both genetic and environmental factors. Third, despite adjusting for multiple confounders like sociodemographic, maternal, and child characteristics, residual confounding from unmeasured covariates may exist.

## Conclusion

We observed no overall association of AD and asthma with delays in communication, gross and fine motor, problem-solving, and personal-social skills. However, children with FA showed a notable association with gross motor delay during the initial 3 years of life. Longitudinal studies with extensive follow-up are crucial for a thorough understanding of the neurodevelopmental effects of these diseases.

## Materials and methods

### Ethical and regulatory considerations

The JECS protocol was reviewed and approved by the Ministry of the Environment's Institutional Review Board on Epidemiological Studies and the Ethics Committees of all participating institutions (No. 100910001). This study adhered to the tenets of the Declaration of Helsinki and Ethical Guidelines for Medical and Health Research Involving Human Subjects, established by the Ministry of Education, Culture, Sports, Science and Technology and Ministry of Health, Labour, and Welfare. Written informed consent was obtained from all participants. Additionally, we followed the Strengthening the Reporting of Observational Studies in Epidemiology (STROBE) reporting guidelines.

### Study design, data sources, and participants

The JECS design has been previously described in detail^48^. Briefly, initiated in 2011, this nationwide prospective birth cohort study aims to evaluate the influence of environmental factors on the health and developmental outcomes of children^48,49^. Fifteen Regional Centers across Japan collaborated on this project, which observes children from prenatal stages up to 13 years of age. To qualify for participation in this study, individuals had to be pregnant and living within the designated study area during the period from January 2011 to March 2014. Of the initial 104,062 fetal records, 100,303 live births were included in the analysis. Exclusions were made for cases involving multiple births, preterm delivery, low birth weight, congenital anomalies, as well as missing and indeterminate child’s sex. Figure 1 provides an overview of the patients’ inclusion; the final sample size consisted of 87,986 children. Data were sourced from the jecs-ta-20190930-qsn dataset, released in October 2019.

### Exposure

In this study, the exposures of interest were caregiver-reported physician-diagnosed allergic diseases, specifically AD, asthma, and FA. Participants provided information on their child's physician diagnoses of these allergic diseases through self-administered questionnaires conducted at 1, 1.5, 2, and 3 years of age, which included specific inquiries about diagnoses within the past 12 months. The questionnaire includes “Has your child ever been diagnosed with the following allergic diseases (a list of allergic conditions was provided in the questionnaire) by a physician in the past 12 months?” This includes instances where the child continues to visit the hospital or receive treatment. In this study, we primarily defined specific allergic conditions based on a caregiver’s affirmative response to the questions at any survey point between a child’s age of 1 and 3 years. We then categorized the persistence of allergies based on the frequency of reported allergies as follows: persistent (allergy reported at all survey points), intermittent (allergy reported at one, two, or three survey points), and none (no allergy reported at any survey point).

Furthermore, we incorporated caregiver-reported allergic features, specifically eczema/AD and wheezing features, utilizing a modified questionnaire adapted from the ISAAC^50–52^, with a validated Japanese translation (Supplementary Table S10).

### Developmental assessment

Between 1 and 3 years of age, we evaluated the neurodevelopmental progress of the children using the Japanese version of the ASQ-3^53^, sent to the parents/caregivers by post every six months postpartum. The ASQ-3, a parental-reported developmental screening tool, is widely used in clinical settings and research and has high reliability and validity^53,54^. It examines five domains: communication, gross motor, fine motor, problem-solving, and personal-social skills (Methods, Supplementary content). To measure the achievement of specific developmental milestones, six questions were asked in each domain; responses of “yes,” “sometimes,” or “not yet” corresponded to scores of 10, 5, and 0, respectively. The sum of the scores of the individual items provided the overall score for each domain (range: 0–60).

In this study, child development was characterized by a cumulative score within each domain falling more than 2 SDs below that of the reference mean labeled as “typical” or “potentially delayed.” The age-specific cutoff values (Supplementary Table S11) were validated by Mezawa et al.^53^, indicating the need for additional assessments of the specific domain in question. A previous study revealed that this threshold demonstrated moderate sensitivity and specificity in detecting delays from mild to severe^55^; moreover, it has been widely employed in the neurodevelopmental assessment of Japanese children^56–58^. In the sensitivity analyses, we additionally utilized an alternative approach by setting the cutoff values to less than 2 SDs for each ASQ-3 domain in the current sample mean.

### Potential confounders

To elucidate the causal pathways in the relationship between exposure and outcomes, we identified potential confounding factors by reviewing the existing literature and conceptualized them within a directed acyclic graph (Supplementary Fig. S1). The following covariates were included: maternal age at delivery, marital status, pre-pregnancy body mass index (expressed as kg/m^2^), gestational diabetes, infertility treatment, psychological distress, iron and folic acid supplementation during pregnancy, pre-pregnancy history of AD, asthma, FA, alcohol consumption during pregnancy, maternal and paternal formal education, smoking during pregnancy, annual household income, mode of delivery, child’s sex, gestational age, birth weight, breastfeeding, formula feeding, and nursery attendance (covariate sources are provided in the Supplementary Methods).

### Statistical analysis

Descriptive statistics were utilized to present the characteristics of the study participants and the developmental status of the children. Our primary focus was to assess the overall association of AD, asthma, and FA with neurodevelopmental milestones measured at multiple time points (1, 1.5, 2, 2.5, and 3 years). To estimate weighted odds ratios (ORs) with 95% CIs, we utilized sIPW logistic regression within a GEE framework. The GEE models were constructed using a binomial probability distribution, logit link function, robust standard error, and an independent working correlation matrix structure.

To obtain the sIPW logistic regression model, we initially calculated the propensity score (PS) of each participant, representing the predicted probability of having specific allergic diseases (cases) versus that of not having them (controls). This was achieved through a multivariable logistic regression model incorporating the previously described covariates. Subsequently, to balance the allergic and non-allergic groups, the estimated PS was used to assign weights to each participant, with scores of 1/PS and 1/(1-PS) being assigned to allergic and non-allergic individuals, respectively. The sIPW logistic regression model was then derived based on the estimated inverse probability weights for all comparisons. The between-group differences characteristics (before and after the weighting) were evaluated using standardized differences with a cutoff of 0.15. The weighted characteristics and standardized mean differences are provided in Supplementary Tables S12 and S13.

Next, we assessed the persistence of allergies between ages 1 and 3 years with neurodevelopmental milestones using logistic regression within GEE models. Additionally, we examined the association between ISAAC-based allergic features (eczema/AD and wheezing) and neurodevelopmental milestones at 3 years of age using sIPW logistic regression models.

To ensure the robustness of the observed associations between allergic diseases and neurodevelopmental delays, the following sensitivity analyses were performed: (a) extreme weights (1st and 99th percentiles) were trimmed in the analyses; (b) multiple imputations were performed for missing values (10 imputed datasets were generated for each variable, and their estimates were combined); (c) excluded ASQ-3 missing values; (d) application of present sample mean—(2 × SD) as cutoff values for each ASQ-3 domain; (e) characterized allergic comorbidities according to the number of reported allergies, and (f) computation of the E-value for neurodevelopmental delays to address any bias caused by unmeasured variables in our primary analysis^59,60^. The E-value serves as a threshold indicating the minimum level of association that an unmeasured confounding factor would need to have to negate the observed relationship and render it insignificant. Furthermore, we examined the potential effect modification by the child’s sex through the inclusion of an interaction term in the GEE model. All analyses were two-tailed (α significance level, 0.05) and were conducted using IBM SPSS Statistics version 25.0 (IBM, Armonk, NY, USA). For subgroup analysis, an α significance level of 0.0033 was considered to avoid multiplicity issues using the Bonferroni correction method.

### Supplementary Information

Supplementary Information.

## Data Availability

Data are unsuitable for public deposition due to ethical restrictions and the legal framework of Japan. It is prohibited by the Act on the Protection of Personal Information (Act No. 57 of 30 May 2003, amendment on 9 September 2015) to publicly deposit data containing personal information. Ethical Guidelines for Medical and Health Research Involving Human Subjects enforced by the Japan Ministry of Education, Culture, Sports, Science and Technology and the Ministry of Health, Labour and Welfare also restricts the open sharing of epidemiologic data. All inquiries about access to data should be sent to jecs-en@nies.go.jp. The person responsible for handling inquiries sent to this e-mail address is Dr Shoji F. Nakayama, JECS Programme Office, National Institute for Environmental Studies.

## Abbreviations

AD: Atopic dermatitis
ADHD: Attention-deficit/hyperactivity disorder
ASD: Autism spectrum disorders
ASQ-3: Ages and Stages Questionnaires, third edition
FA: Food allergy
GEE: Generalized estimating equation
JECS: Japan Environment and Children’s Study
NDDs: Neurodevelopmental disorders
PS: Propensity score
sIPW: Stabilized inverse probability-weighted
