# Supplementary material for: Early childhood neurodevelopmental milestones in children with allergic diseases: the Japan Environment and Children’s Study (JECS)
Source: Sci Rep. 2024 Mar 18;14:6460. doi: 10.1038/s41598-024-57210-y (PMC10948893; doi:10.1038/s41598-024-57210-y)
Supplement: Supplementary file 1 — Supplementary Information. [file 41598_2024_57210_MOESM1_ESM.pdf]

Early childhood neurodevelopmental milestones in children with allergic diseases: the Japan Environment and Children's Study (JECS)

Abir Nagata, PhD[1,2,*]; Kazunari Onishi, MSc, PhD[3,*]; Toshio Masumoto, PhD[4]; Takatoshi Nakagawa, PhD[1]; Kazue Ishitsuka, MD, MPH, PhD[5]; and Youichi Kurozawa, MD, PhD[4]

[1]Department of Regenerative Dermatology, Graduate School of Medicine, Osaka University, Osaka, Japan

[2]Graduate School of Public Health, St. Luke's International University, Tokyo, Japan

[3]Division of Environmental Health, Graduate School of Public Health, St. Luke's International University, Tokyo, Japan

[4]Division of Health Administration and Promotion, Faculty of Medicine, Tottori University, Tottori, Japan

[5]Department of Social Medicine, National Center for Child Health and Development, Tokyo, Japan

*** Corresponding Authors:**

Abir Nagata, PhD

Department of Regenerative Dermatology, Graduate School of Medicine, Osaka University.

2-2, Yamadaoka, Suita -shi, Osaka 565-0871, Japan

E-mail: abir.med@osaka-u.ac.jp

Tell: (81) 6-6879-3960

and

Kazunari Onishi, MSc, PhD

Division of Environmental Health, Graduate School of Public Health, St. Luke's International University, Tokyo, Japan. 3-6-2 Tsukiji, Chuo-ku, Tokyo 104-0045 Japan

E-mail: kaznaly@slcn.ac.jp

Tel: +81-3-5550-4101

Supplementary Material

List of Contents

- **Methods:** Supplementary Methods
- **Table 1.** Frequencies of suspected developmental delay in ASQ-3 milestones (n = 87986)
- **Table 2.** Frequencies of suspected neurodevelopmental delay in ASQ-3 milestones according to the early childhood allergic disorders
- **Table 3.** Association of ISAAC-based eczema/AD and wheezing features with ASQ-3 developmental milestones
- **Table 4.** Sensitivity analysis of the association of allergic diseases with ASQ-3 developmental milestones in trimmed stabilized weighted generalized estimating equations (GEEs) models
- **Table 5.** Sensitivity analysis of the association of allergic diseases with ASQ-3 developmental milestones in GEE models with multiple imputation
- **Table 6**. Sensitivity analysis of the association of allergic diseases with ASQ-3 developmental milestones in GEE models, excluding ASQ-3 missing values (n = 53,364)
- **Table 7.** Sensitivity analysis of the association of allergic diseases with ASQ-3 developmental milestones with sample mean – (2 × standard deviation [SD]) as cutoff values for each J-ASQ-3 domain
- **Table 8.** Association of allergic comorbidities with ASQ-3 developmental milestones.
- **Table 9.** Subgroup analysis by child's sex for the association of early childhood allergic diseases with ASQ-3 developmental milestones
- **Table 10.** Definitions of caregiver-reported ISAAC-based allergic features
- **Table 11.** Age-specific validated ASQ-3 cutoff values
- **Table 12.** Characteristics of the attained stabilized weights
- **Table 13.** Covariate balance between allergic and non-allergic groups
- **Figure 1.** Directed acyclic graph for the association between early childhood allergic diseases and neurodevelopment
- **References**

Supplementary Methods

Developmental Assessment (ASQ-3 questionnaire)

Communication Skills: This encompasses the ability to ask questions, communicate clearly, build friendships, and share personal information.

Gross Motor Skills: These encompass coordination, balance, strength, spatial awareness, and rapid reactions.

Fine Motor Skills: This pertains to the use of tools including scissors, keyboards, and rulers, as well as tasks such as holding a pen, drawing, and writing neatly.

Problem-Solving: It involves creativity, decision-making, reliability, and effective teamwork.

Personal Skills: These encompass dependability, adaptability, and motivation.

Social Skills: These includes aspects such as sharing, cooperation, maintaining eye contact, active listening, respecting personal boundaries, and displaying good manners.

Potential Confounders

Maternal age at delivery (<25, 25–29, 30–34, and ≥35 years), pre-pregnancy body mass index (<18.5, 18.5–24.9, and ≥25 kg/m^2), administration of infertility treatment (yes/no), gestational diabetes (yes/no), iron and folic acid supplementation during pregnancy (yes/no), gestational weeks (37–38 and 39–41 weeks), mode of delivery (cesarean and vaginal), child's sex (male and female), and birth weight categories (2500–4000 g and >4000 g) were extracted from medical records. Information on maternal marital status (single, married, and divorced/widowed), history of maternal and paternal smoking during pregnancy (yes/no), history of maternal alcohol consumption during pregnancy (yes/no), maternal and paternal education (high school education or lower, college education, and bachelor's degree or higher), maternal history of pre-pregnancy allergic diseases (atopic dermatitis, asthma, and food allergy), annual household income (<4, 4–8, and >8 million Japanese yen), maternal psychological distress assessed using the K6 scale during pregnancy (1–4 and ≥5), child's nursery school attendance at 1 year (yes/no), and breastfeeding/formula feeding duration (1–6 and 7–12 months) were obtained through self-administered questionnaires. The Japanese version of the K6 scale was employed to assess depressive tendencies, and a cutoff value of 5 points or higher was used [1]. A K6 score exceeding the cutoff (≥5 points) indicated poor mental health.

Table 1. Frequencies of suspected developmental delay in ASQ-3 milestones (n = 87,986)

Child's age	Development status	ASQ-3 subscale				
		**Communication n (%)**	**Gross motor n (%)**	**Fine motor n (%)**	**Problem-solving n (%)**	**Personal social n (%)**
1 year	Typical	72147 (82.0)	68341 (77.7)	68167 (77.5)	68538 (77.9)	71201 (80.9)
	Delayed	74 (0.1)	3883 (4.4)	4021 (4.5)	3580 (4.1)	796 (0.9)
	Missing data	15765 (17.9)	15762 (17.9)	15798 (18.0)	15868 (18.0)	15989 (18.2)
1.5 years	Typical	66689 (75.8)	65145 (74.0)	65246 (74.2)	65042 (73.9)	68699 (78.1)
	Delayed	1386 (1.6)	2947 (3.4)	2809 (3.1)	2622 (3.0)	941 (1.1)
	Missing data	19911 (22.6)	19894 (22.6)	19931 (22.7)	20322 (23.1)	18346 (20.8)
2 years	Typical	67176 (76.4)	65920 (74.9)	68297 (77.6)	66799 (75.9)	67814 (77.1)
	Delayed	2559 (2.9)	3819 (4.3)	1379 (1.6)	2746 (3.1)	1826 (2.1)
	Missing data	18251 (20.7)	18247 (20.8)	18310 (20.8)	18441 (21.0)	18346 (20.8)
2.5 years	Typical	64897 (73.8)	65335 (74.3)	63980 (72.7)	64206 (73.0)	65826 (74.8)
	Delayed	3139 (3.5)	2722 (3.1)	3754 (4.3)	3673 (4.2)	2114 (2.4)
	Missing data	19950 (22.7)	19929 (22.6)	20252 (23.0)	20107 (22.8)	20046 (22.8)
3 years	Typical	67076 (76.2)	66859 (76.0)	64466 (73.3)	64267 (73.0)	67475 (76.7)
	Delayed	2551 (2.9)	2866 (3.3)	4993 (5.6)	4803 (5.5)	2082 (2.4)
	Missing data	18359 (20.9)	18261 (20.7)	18527 (21.1)	18916 (21.5)	18429 (20.9)

Abbreviations: ASQ-3, Ages and Stages Questionnaires, third edition
Child neurodevelopment was assessed using a total score in each domain that fell less than 2 SDs below the mean in the reference, denoted as either typical or potentially delayed. The cutoff values for ASQ-3 are detailed in Table 11.

Table 2. Frequencies of suspected neurodevelopmental delay in ASQ-3 milestones according to the early childhood allergic diseases

Child's age	Allergic disorders	ASQ-3 subscale									
		Communication		Gross motor		Fine motor		Problem-solving		Personal social	
		Delayed	Typical	Delayed	Typical	Delayed	Typical	Delayed	Typical	Delayed	Typical
		n (%)	n (%)	n (%)	n (%)	n (%)	n (%)	n (%)	n (%)	n (%)	n (%)
**1 year**	**Atopic dermatitis**										
	Yes	7 (0.1)	8132 (99.9)	442 (5.4)	7699 (94.6)	460 (5.7)	7674 (94.3)	408 (5.0)	7718 (95.0)	86 (1.1)	8027 (98.9)
	No	67 (0.1)	64015 (99.9)	3441 (5.4)	60642 (94.6)	3561 (5.6)	60493 (94.4)	3172 (5.0)	60820 (95.0)	710 (1.1)	63174 (98.9)
	**Asthma**										
	Yes	8 (0.1)	7891 (99.9)	423 (5.4)	7477 (94.6)	461 (5.8)	7435 (94.2)	390 (4.9)	7500 (95.1)	96 (1.2)	7781 (98.8)
	No	66 (0.1)	44256 (99.9)	3459 (5.4)	60864 (94.6)	3560 (5.5)	60732 (94.5)	3190 (5.0)	61038 (95.0)	700 (1.1)	63420 (98.9)
	**Food allergy**										
	Yes	8 (0.1)	10519 (99.9)	610 (5.8)	9917 (94.2)	598 (5.7)	9921 (94.3)	544 (5.2)	9964 (94.8)	133 (1.3)	10364 (98.7)
	No	66 (0.1)	61628 (99.9)	3273 (5.3)	58424 (94.7)	3432 (5.6)	58246 (94.4)	3036 (4.9)	58574 (95.1)	663 (1.1)	60837 (98.9)
**1.5 years**	**Atopic dermatitis**										
	Yes	157 (2.0)	7614 (98.0)	342 (4.4)	7433 (95.6)	298 (3.8)	7471 (96.2)	307 (4.0)	7419 (96.0)	92 (1.2)	7898 (98.8)
	No	1229 (2.0)	59075 (98.0)	2605 (4.3)	57712 (95.7)	2511 (4.2)	57775 (95.8)	2315 (3.9)	57623 (96.1)	849 (1.4)	60801 (98.6)
	**Asthma**										
	Yes	110 (1.5)	7429 (98.5)	303 (4.0)	7239 (96.0)	273 (3.6)	7264 (96.4)	273 (3.6)	7235 (96.4)	68 (0.9)	7727 (99.1)

**Child's age**	**Allergic disorders**	**ASQ-3 subscale**									
		**Communication**		**Gross motor**		**Fine motor**		**Problem-solving**		**Personal social**	
		**Delayed**	**Typical**	**Delayed**	**Typical**	**Delayed**	**Typical**	**Delayed**	**Typical**	**Delayed**	**Typical**
		**n (%)**	**n (%)**	**n (%)**	**n (%)**	**n (%)**	**n (%)**	**n (%)**	**n (%)**	**n (%)**	**n (%)**
	No	1276 (2.1)	59260 (97.9)	2644 (4.4)	57906 (95.6)	2536 (4.2)	57982 (95.8)	2349 (3.9)	57807 (96.1)	873 (1.4)	60972 (98.6)
	**Food allergy**										
	Yes	204 (2.0)	9826 (98.0)	471 (4.7)	9562 (95.3)	378 (3.8)	9648 (96.2)	382 (3.8)	9591 (96.2)	136 (1.3)	10204 (98.7)
	No	1182 (2.0)	56863 (98.0)	2476 (4.3)	55583 (95.7)	2431 (4.2)	55598 (95.8)	2240 (3.9)	55451 (96.1)	805 (1.4)	58495 (98.6)
**2 years**	**Atopic dermatitis**										
	Yes	289 (3.6)	7711 (96.4)	426 (5.3)	7575 (94.7)	147 (1.8)	7846 (98.2)	292 (3.7)	7687 (96.3)	211 (2.6)	7779 (97.4)
	No	2270 (3.7)	59465 (96.3)	3393 (5.5)	58345 (94.5)	1232 (2.0)	60451 (98.0)	2454 (4.0)	59112 (96.0)	1615 (2.6)	60035 (97.4)
	**Asthma**										
	Yes	244 (3.1)	7563 (96.9)	375 (4.8)	7430 (95.2)	151 (1.9)	7647 (98.1)	266 (3.4)	7521 (96.6)	177 (2.3)	7618 (97.7)
	No	2315 (3.7)	59613 (96.3)	3444 (5.6)	58490 (94.4)	1228 (2.0)	60650 (98.0)	2480 (4.0)	59278 (96.0)	1649 (2.7)	60196 (97.3)
	**Food allergy**										
	Yes	395 (3.8)	9957 (96.2)	582 (5.6)	9768 (94.4)	207 (2.0)	10134 (98.0)	423 (4.1)	9897 (95.9)	289 (2.8)	10051 (97.2)
	No	2164 (3.6)	57219 (96.4)	3237 (5.5)	56152 (94.5)	1172 (2.0)	58163 (98.0)	2323 (3.9)	56902 (96.1)	1537 (2.6)	57763 (97.4)
**2.5 years**	**Atopic dermatitis**										
	Yes	344 (4.4)	7427 (95.6)	319 (4.1)	7457 (95.9)	447 (5.8)	7284 (94.2)	436 (5.6)	7321 (94.4)	244 (3.1)	7517 (96.9)

Child's age	Allergic disorders	ASQ-3 subscale									
		Communication		Gross motor		Fine motor		Problem-solving		Personal social	
		Delayed	Typical	Delayed	Typical	Delayed	Typical	Delayed	Typical	Delayed	Typical
		n (%)	n (%)	n (%)	n (%)	n (%)	n (%)	n (%)	n (%)	n (%)	n (%)
	No	2795 (4.6)	57470 (95.4)	2403 (4.0)	57878 (96.0)	3307 (5.5)	56696 (94.5)	3237 (5.4)	56885 (94.6)	1870 (3.1)	58309 (96.9)
	**Asthma**										
	Yes	317 (4.2)	7259 (95.8)	306 (4.0)	7268 (96.0)	406 (5.4)	7143 (94.6)	378 (5.0)	7180 (95.0)	210 (2.8)	7353 (97.2)
	No	2822 (4.7)	57638 (95.3)	2416 (4.0)	58067 (96.0)	3348 (5.6)	56837 (94.4)	3295 (5.5)	57026 (94.5)	1904 (3.2)	58473 (96.8)
	**Food allergy**										
	Yes	455 (4.5)	9643 (95.5)	460 (4.6)	9637 (95.4)	578 (5.8)	9456 (94.2)	553 (5.5)	9520 (94.5)	321 (3.2)	9758 (96.8)
	No	2684 (4.6)	55254 (95.4)	2262 (3.9)	55698 (96.1)	3176 (5.5)	54524 (94.5)	3120 (5.4)	54686 (94.6)	1793 (3.1)	56068 (96.9)
**3 years**	**Atopic dermatitis**										
	Yes	293 (3.6)	7737 (96.4)	331 (4.1)	7712 (95.9)	615 (7.7)	7401 (92.3)	576 (7.2)	7394 (92.8)	247 (3.1)	7783 (96.9)
	No	2258 (3.7)	59339 (96.3)	2535 (4.1)	59147 (95.9)	4378 (7.1)	57065 (92.9)	4227 (6.9)	56873 (93.1)	1835 (3.0)	59692 (97.0)
	**Asthma**										
	Yes	264 (3.3)	7654 (96.7)	334 (4.2)	7591 (95.8)	629 (8.0)	7268 (92.0)	514 (6.6)	7333 (93.4)	228 (2.9)	7675 (97.1)
	No	2287 (3.7)	59422 (96.3)	2532 (4.1)	59268 (95.9)	4364 (7.1)	57198 (92.9)	4289 (7.0)	56934 (93.0)	1854 (3.0)	59800 (97.0)
	**Food allergy**										
	Yes	364 (3.5)	9992 (96.5)	442 (4.3)	9920 (95.7)	787 (7.6)	9543 (92.4)	704 (6.9)	9572 (93.1)	311 (3.0)	10028 (97.0)

Child's age	Allergic disorders	ASQ-3 subscale									
		Communication		Gross motor		Fine motor		Problem-solving		Personal social	
		Delayed	Typical	Delayed	Typical	Delayed	Typical	Delayed	Typical	Delayed	Typical
		n (%)	n (%)	n (%)	n (%)	n (%)	n (%)	n (%)	n (%)	n (%)	n (%)
	No	2187 (3.7)	57084 (96.3)	2424 (4.1)	56939 (95.9)	4206 (7.1)	54923 (92.9)	4099 (7.0)	54695 (93.0)	1771 (3.0)	57447 (97.0)

Cutoff values of ASQ-3 are provided in supplementary material (Table 11).
ASQ-3, Ages and Stages Questionnaires, third edition

Table 3. Association of ISAAC-based eczema/AD and wheezing features with ASQ-3 developmental milestones

Allergic features	ASQ-3 domains, weighted AOR (95% CI)[a]				
**Eczema/AD[b]**	**Communication skills**	**Gross Motor skills**	**Fine Motor skills**	**Problem-solving skills**	**Personal-social skills**
No	1 [Reference]	1 [Reference]	1 [Reference]	1 [Reference]	1 [Reference]
Yes	0.99 (0.85–1.13)	1.00 (0.89–1.12)	1.04 (0.91–1.10)	0.95 (0.89–1.07)	0.97 (0.84–1.08)
**Wheezing[c]**					
No	1 [Reference]	1 [Reference]	1 [Reference]	1 [Reference]	1 [Reference]
Yes	0.84 (0.75–1.01)	1.02 (0.95–1.10)	0.98 (0.88–1.05)	0.93 (0.82–1.03)	0.94 (0.85–1.06)

Abbreviations: International Study of Asthma and Allergies in Childhood; AOR, adjusted odds ratio; CI, confidence interval; ASQ-3, Ages and Stages Questionnaires, third edition.
[a] Weighted AOR were derived via propensity score based, logistic regression models.
[b] Adjusted for wheezing features.
[c] Adjusted for eczema/AD features

Table 4. Sensitivity analysis of the association of allergic diseases with ASQ-3 developmental milestones in trimmed stabilized weighted generalized estimating equations (GEEs) models

**Allergic diseases**	**ASQ-3 domains, weighted AOR (95% CI)**[a]				
**Atopic dermatitis**[b]	**Communication skills**	**Gross Motor skills**	**Fine Motor skills**	**Problem-solving skills**	**Personal-social skills**
No	1 [Reference]	1 [Reference]	1 [Reference]	1 [Reference]	1 [Reference]
Yes	1.03 (0.86–1.15)	0.98 (0.88–1.06)	1.03 (0.93–1.11)	1.07 (0.95–1.18)	0.98 (0.84–1.10)
**Asthma**[c]					
No	1 [Reference]	1 [Reference]	1 [Reference]	1 [Reference]	1 [Reference]
Yes	0.80 (0.70–0.91)[e]	1.02 (0.91–1.10)	0.97 (0.90–1.06)	0.95 (0.86–1.03)	0.94 (0.85–1.03)
**Food allergy**[d]					
No	1 [Reference]	1 [Reference]	1 [Reference]	1 [Reference]	1 [Reference]
Yes	0.96 (0.85–1.10)	1.17 (1.07–1.28)[e]	1.05 (0.96–1.14)	1.05 (0.97–1.15)	1.02 (0.91–1.15)

Abbreviations: AOR, adjusted odds ratio; CI, confidence interval; ASQ-3, Ages and Stages Questionnaires, third edition.
[a] Weighted AOR was derived via propensity score-based, stabilized inverse probability weighted GEE models.
[b] adjusted for asthma and food allergy
[c] adjusted for atopic dermatitis and food allergy
[d] adjusted for asthma and atopic dermatitis
[e] $p<0.001$

Table 5. Sensitivity analysis of the association of allergic diseases with ASQ-3 developmental milestones in GEE models with multiple imputation

**Allergic diseases**	**ASQ-3 domains, weighted AOR (95% CI)[a]**				
**Atopic dermatitis[b]**	**Communication skills**	**Gross Motor skills**	**Fine Motor skills**	**Problem-solving skills**	**Personal-social skills**
No	1 [Reference]	1 [Reference]	1 [Reference]	1 [Reference]	1 [Reference]
Yes	0.95 (0.80–1.11)	0.98 (0.85–1.06)	1.03 (0.93–1.10)	1.03 (0.92–1.09)	0.96 (0.85–1.08)
**Asthma[c]**					
No	1 [Reference]	1 [Reference]	1 [Reference]	1 [Reference]	1 [Reference]
Yes	0.80 (0.71–0.93)[e]	1.06 (0.93–1.12)	0.96 (0.87–1.09)	0.92 (0.84–1.04)	0.93 (0.84–1.05)
**Food allergy[d]**					
No	1 [Reference]	1 [Reference]	1 [Reference]	1 [Reference]	1 [Reference]
Yes	1.01 (0.89–1.11)	1.11 (1.04–1.25)[e]	1.05 (0.95–1.08)	1.01 (0.94–1.06)	1.01 (0.92–1.13)

Abbreviations: AOR, adjusted odds ratio; CI, confidence interval; ASQ-3, Ages and Stages Questionnaires, third edition.

[a] Weighted AOR was derived via propensity score-based, stabilized inverse probability weighted GEE models.

[b] adjusted for asthma and food allergy

[c] adjusted for atopic dermatitis and food allergy

[d] adjusted for asthma and atopic dermatitis

[e] $p<0.001$

Table 6. Sensitivity analysis of the association of allergic diseases with ASQ-3 developmental milestones in GEE models, excluding missing ASQ-3 values (n = 53,364)

**Allergic diseases**	**ASQ-3 domains, weighted AOR (95% CI)[a]**				
**Atopic dermatitis[b]**	**Communication skills**	**Gross Motor skills**	**Fine Motor skills**	**Problem-solving skills**	**Personal-social skills**
No	1 [Reference]	1 [Reference]	1 [Reference]	1 [Reference]	1 [Reference]
Yes	1.02 (0.89–1.16)	0.99 (0.88–1.06)	1.03 (0.91–1.08)	1.03 (0.93–1.09)	0.92 (0.81–1.06)
**Asthma[c]**					
No	1 [Reference]	1 [Reference]	1 [Reference]	1 [Reference]	1 [Reference]
Yes	0.83 (0.72–0.95)[e]	0.98 (0.90–1.12)	0.98 (0.92–1.06)	0.94 (0.88–1.01)	0.90 (0.81–1.03)
**Food allergy[d]**					
No	1 [Reference]	1 [Reference]	1 [Reference]	1 [Reference]	1 [Reference]
Yes	0.95 (0.80–1.09)	1.12 (1.02–1.24)[e]	1.04 (0.96–1.09)	1.03 (0.95–1.08)	1.01 (0.92–1.11)

Abbreviations: AOR, adjusted odds ratio; CI, confidence interval; ASQ-3, Ages and Stages Questionnaires, third edition.

[a] Weighted AOR was derived via propensity score-based, stabilized inverse probability weighted GEE models.

[b] adjusted for asthma and food allergy

[c] adjusted for atopic dermatitis and food allergy

[d] adjusted for asthma and atopic dermatitis

[e] $p<0.01$

Table 7. Sensitivity analysis on the association of allergic diseases with ASQ-3 developmental milestones with sample mean – (2 × standard deviation [SD]) as cutoff values for each ASQ-3 domain

**Allergic diseases**	**ASQ-3 domains, weighted AOR (95% CI)[a]**				
**Atopic dermatitis[b]**	**Communication skills**	**Gross Motor skills**	**Fine Motor skills**	**Problem-solving skills**	**Personal-social skills**
No	1 [Reference]	1 [Reference]	1 [Reference]	1 [Reference]	1 [Reference]
Yes	1.01 (0.89–1.18)	0.93 (0.81–1.07)	1.05 (0.94–1.11)	0.99 (0.90–1.12)	0.98 (0.85–1.10)
**Asthma[c]**					
No	1 [Reference]	1 [Reference]	1 [Reference]	1 [Reference]	1 [Reference]
Yes	0.91 (0.80–0.95)[e]	1.00 (0.91–1.06)	0.96 (0.88–1.07)	0.93 (0.88–1.05)	0.96 (0.84–1.07)
**Food allergy[d]**					
No	1 [Reference]	1 [Reference]	1 [Reference]	1 [Reference]	1 [Reference]
Yes	1.01 (0.90–1.11)	1.10 (1.03–1.17)[e]	1.02 (0.93–1.08)	1.04 (0.95–1.09)	1.01 (0.90–1.13)

Abbreviations: AOR, adjusted odds ratio; CI, confidence interval; ASQ-3, Ages and Stages Questionnaires, third edition.

[a] Weighted AOR was derived via propensity score-based, stabilized inverse probability weighted GEE models.

[b] adjusted for asthma and food allergy

[c] adjusted for atopic dermatitis and food allergy

[d] adjusted for asthma and atopic dermatitis

[e] $p<0.01$

Table 8. Association of allergic comorbidities with ASQ-3 developmental milestones

**Allergic comorbidities**	**Child, No. (%)**	**ASQ-3 domains, AOR (95% CI)[a]**				
		**Communication skills**	**Gross motor skills**	**Fine motor skills**	**Problem-solving skills**	**Personal-social skills**
**No allergy[b]**	66750 (75.9)	1 [Reference]	1 [Reference]	1 [Reference]	1 [Reference]	1 [Reference]
**Any allergy**	13668 (15.5)	0.91 (0.82–1.00)	1.03 (0.97–1.10)	1.10 (0.95–1.27)	0.96 (0.82–1.12)	0.85 (0.62–1.17)
**AD + Asthma**	574 (0.6)	1.06 (0.68–1.63)	1.10 (0.84–1.43)	1.10 (0.95–1.27)	0.96 (0.82–1.12)	0.85 (0.62–1.17)
**AD + FA**	4807 (5.5)	1.02 (0.88–1.18)	1.01 (0.92–1.11)	1.05 (0.92–1.19)	1.08 (0.95–1.12)	0.96 (0.73–1.27)
**Asthma + FA**	908 (1.0)	0.99 (0.70–1.38)	1.37 (1.13–1.66)[d]	1.11 (0.96–1.27)	1.13 (0.98–1.29)	1.25 (0.95–1.64)
**AD + Asthma + FA**	1279 (1.5)	1.02 (0.77–1.36)	1.20 (1.01–1.43)[c]	1.10 (0.95–1.27)	0.96 (0.82–1.12)	0.85 (0.62–1.17)

Abbreviations: AD, atopic dermatitis; FA, food allergy; AOR, adjusted odds ratio; CI, confidence interval; ASQ-3, Ages and Stages Questionnaires, third edition.

[a] AOR was obtained via generalized estimating equations models (adjusted for maternal age at delivery, marital status, pre-pregnancy body mass index, gestational diabetes, infertility treatment, psychological distress, iron and folic acid supplementation, pre-pregnancy history of AD, asthma, FA, alcohol consumption, maternal and paternal formal education, smoking during pregnancy, household income, mode of delivery, child's sex, gestational age, birth weight, breastfeeding, formula feeding, and child’s nursery attendance)

[b] Participants with no reported allergic conditions at any survey point.

[c] $p<0.05$; [d] $p<0.001$

Table 9. Subgroup analysis by child's sex for the association of early childhood allergic diseases with ASQ-3 developmental milestones

**Allergic diseases**		**Boys**	**Girls**	
	ASQ	Weighted AOR (95% CI)	Weighted AOR (95% CI)	*P* for interaction
Atopic dermatitis[a]	Communication	1.06 (0.92–1.21)	0.89 (0.75–1.12)	.051
	Gross Motor	1.03 (0.91–1.16)	0.99 (0.87–1.12)	.681
	Fine Motor	1.03 (0.93–1.13)	1.02 (0.89–1.16)	.904
	Problem-solving	1.04 (0.95–1.15)	0.96 (0.95–1.15)	.332
	Personal social	1.01 (0.88–1.17)	0.68 (0.51–1.01)	.049
Asthma[b]	Communication	01.01 (0.90–1.10)	0.75 (0.62–1.01)	.091
	Gross Motor	01.01 (0.87–1.07)	1.05 (0.92–1.18)	.294
	Fine Motor	0.99 (0.87–1.07)	1.01 (0.85–1.20)	.266
	Problem solving	0.97 (0.85–1.05)	0.99 (0.86–1.09)	.782
	Personal social	0.98 (0.77–1.08)	0.96 (0.75–1.11)	.860
Food allergy[c]	Communication	0.99 (0.88–1.12)	0.79 (0.67–1.01)	.0533
	Gross Motor	1.14 (1.00–1.23)	1.13 (1.01–1.25)	.822
	Fine Motor	1.01 (0.93–1.10)	1.06 (0.94–1.19)	.543
	Problem-solving	1.01 (0.92–1.10)	1.01 (0.90–1.13)	.971
	Personal social	1.03 (0.91–1.17)	0.92 (0.73–1.15)	.374

Data are expressed as weighted adjusted ORs. Weighted AOR was derived via propensity score-based, stabilized inverse probability weighted GEE models.

[a] adjusted for asthma and food allergy

[b] adjusted for atopic dermatitis and food allergy

[c] adjusted for asthma and food allergy

Abbreviations: AOR, adjusted odds ratio; CI, confidence interval; ASQ-3, Ages and Stages Questionnaires, third edition

Table 10. Definitions of caregiver-reported ISAAC-based allergic features

**Allergic features[a]**	**Definition**
Eczema/AD	A positive answer to both of the following questions:<br>1) "Has your child had this itchy rash at any time in the past 12 months?"<br>2) "Has this itchy rash at any time been observed in any of the following places: the folds of the elbows, behind the knees, in front of the ankles, under the buttocks, or around the neck, ears, or eyes?"
Wheezing	A positive answer to both of the following questions:<br>1) "Has your child ever had wheezing or whistling in the chest at any time in the past?"<br>2) "Has your child had wheezing or whistling in the chest in the past 12 months?"
[a] Caregiver-reported wheezing, eczema/AD, and rhinitis symptoms were evaluated using the questionnaire of the International Study of Asthma and Allergies in Childhood (ISAAC) [2] when the child attained the age of 2 and 3 years.	

Table 11. Age-specific validated ASQ-3 cutoff values

**ASQ-3 subscale**					
Child age	Communication	Gross motor	Fine motor	Problem-solving	Personal social
1 year	4.53	9.43	25.47	15.37	4.95
1.5 years	5.82	37.59	26.76	15.93	24.57
2 years	14.33	39.13	33.48	29.38	25.22
2.5 years	26.01	38.36	21.03	25.78	29.70
3 years	29.95	39.26	27.91	30.03	29.89
Abbreviations: ASQ-3, Ages and Stages Questionnaires, third edition					

Table 12. Characteristics of the attained stabilized weights

**Allergic conditions**	**Estimated weights**	
	Mean (SD)	Min–Max
Atopic dermatitis	0.99 (0.12)	0.27–1.73
Asthma	1.00 (0.15)	0.21–2.58
Food allergy	0.99 (0.14)	0.27–2.61

Table 13. Covariate balance between allergic and non-allergic groups

Variables	Standardized difference					
	**Atopic dermatitis**		**Asthma**		**Food allergy**	
	Before weighting	After weighing	Before weighting	After weighing	Before weighting	After weighing
Age at delivery	-0.004	0.002	0.071	0.000	0.003	0.001
Marital status	0.013	0.001	-0.028	0.007	0.037	0.003
Education	-0.061	-0.003	0.061	0.012	-0.120	-0.004
Pre-pregnancy body mass index (BMI)	0.017	0.006	-0.039	0.006	0.039	0.005
Gestational diabetes	-0.003	0.001	-0.003	0.002	0.011	0.002
Infertility treatment	0.007	-0.002	0.020	-0.007	-0.045	0.000
Psychological distress (K6 scale)	-0.067	-0.001	-0.073	0.004	-0.042	-0.005
Iron pill during pregnancy	0.008	0.010	-0.021	-0.002	-0.006	0.002
Folic acid during pregnancy	0.007	0.003	0.020	-0.002	-0.003	0.003
Smoking during pregnancy	0.019	0.001	-0.080	0.006	0.046	-0.000
Alcohol during pregnancy	0.006	-0.001	-0.015	0.004	0.021	-0.000
Maternal atopic dermatitis	-0.384	-0.004	-0.069	-0.009	-0.282	-0.005
Maternal asthma	-0.137	-0.002	-0.323	-0.000	-0.124	0.002
Maternal food allergy	-0.147	-0.003	-0.094	0.004	-0.155	-0.000
Paternal education	-0.016	0.001	0.100	-0.007	-0.083	-0.003
Paternal smoking	0.025	0.004	-0.068	0.006	0.068	0.001
Income (million Japanese yen/year)	-0.003	-0.008	0.070	-0.011	-0.041	0.001
Mode of delivery	0.006	0.001	-0.021	0.002	0.025	0.002
Child’s sex	0.138	0.000	0.202	0.016	0.162	0.001
Gestational age (weeks)	0.006	0.005	0.070	0.001	-0.046	0.000
Birth weight	-0.027	0.004	-0.012	0.002	-0.016	-0.001
Breastfeeding, 0–6 months	0.021	-0.003	-0.060	0.010	0.092	-0.004
Breastfeeding, 7–12 months	0.058	0.006	-0.136	0.009	0.181	0.005
Formula feeding 0–6 months	-0.078	-0.002	0.102	-0.010	-0.201	-0.002
Formula feeding, 7–12 months	-0.054	-0.006	0.161	-0.008	-0.162	-0.006
Nursery attendance at 1 year	0.083	0.006	0.348	0.004	0.058	0.003

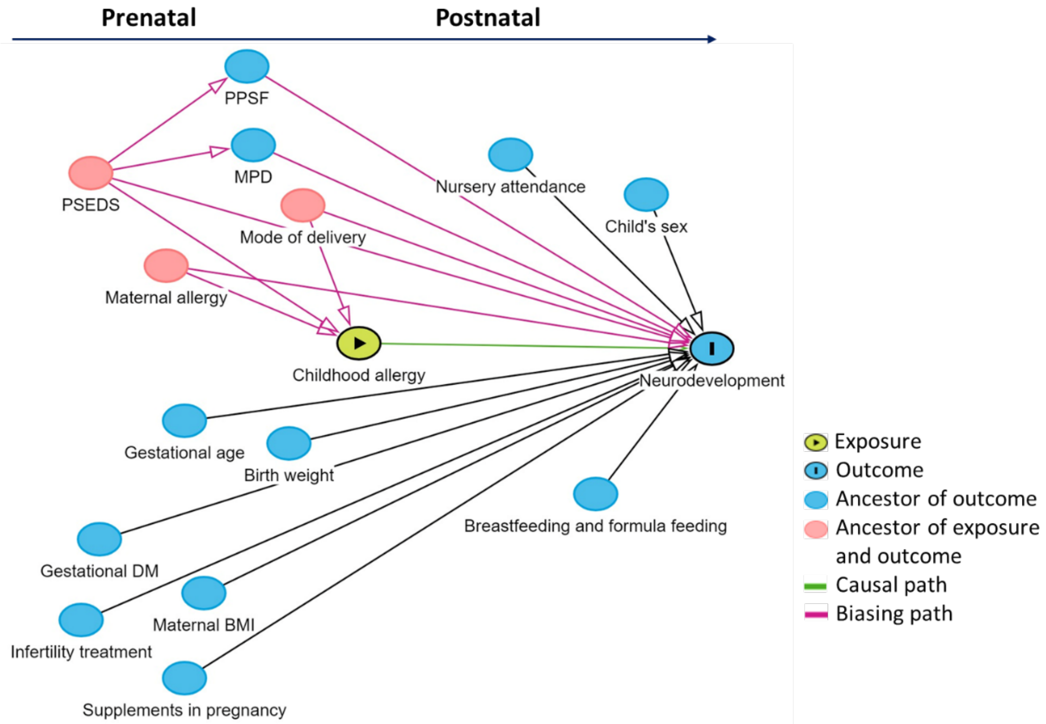

Figure 1. Directed acyclic graph for the association between early childhood allergic diseases and neurodevelopment

Directed acyclic graphs depict the hypothesized potential confounding factors (red nodes) in the association between early childhood allergic diseases and neurodevelopment. Blue nodes: variables associated with neurodevelopment in the offspring. Mediators are not included.

PSEDS, parental socioeconomic and demographic status (maternal age, marital status, maternal and paternal education, and annual household income); PPSF, parental psychosocial factors (maternal and paternal smoking and maternal alcohol consumption during pregnancy); BMI, body mass index (pre-pregnancy); DM; diabetes mellitus; MPD, maternal psychological distress during pregnancy; maternal pre-pregnancy history of allergic diseases; atopic dermatitis, asthma, and food allergy; supplements during pregnancy: iron pill and folic acid.

References

1. Furukawa, T. A., et al. The performance of the Japanese version of the K6 and K10 in the World Mental Health Survey Japan. *Int. J. Meth. Psych. Res.* **17**, 152-158 (2008).

2. Asher, M. I., et al. International Study of Asthma and Allergies in Childhood (Isaac) - Rationale and Methods. *Eur. Respir. J.* **8**, 483-491 (1995).
